# Insecticide resistance and nutrition interactively shape life-history parameters in German cockroaches

**DOI:** 10.1038/srep28731

**Published:** 2016-06-27

**Authors:** Kim Jensen, Alexander E. Ko, Coby Schal, Jules Silverman

**Affiliations:** 1 Department of Entomology, North Carolina State University, Raleigh, NC 27695, USA; 2 W. M. Keck Center for Behavioral Biology, North Carolina State University, Raleigh, NC 27695, USA

## Abstract

Fitness-related costs of evolving insecticide resistance have been reported in a number of insect species, but the interplay between evolutionary adaptation to insecticide pressure and variable environmental conditions has received little attention. We provisioned nymphs from three German cockroach (*Blattella germanica* L.) populations, which differed in insecticide resistance, with either nutritionally rich or poor (diluted) diet throughout their development. One population was an insecticide-susceptible laboratory strain; the other two populations originated from a field-collected indoxacarb-resistant population, which upon collection was maintained either with or without further selection with indoxacarb. We then measured development time, survival to the adult stage, adult body size, and results of a challenge with indoxacarb. Our results show that indoxacarb resistance and poor nutritional condition increased development time and lowered adult body size, with reinforcing interactions. We also found lower survival to the adult stage in the indoxacarb-selected population, which was exacerbated by poor nutrition. In addition, nutrition imparted a highly significant effect on indoxacarb susceptibility. This study exemplifies how poor nutritional condition can aggravate the life-history costs of resistance and elevate the detrimental effects of insecticide exposure, demonstrating how environmental conditions and resistance may interactively impact individual fitness and insecticide efficacy.

Insecticide resistance, a heritable adaptation to insecticide pressure, provides a valuable system to study the evolutionary ecology of adaptation to severe environmental hazards and the consequences to fitness-related life-history parameters within the adapted population. The evolution of insecticide resistance is often accompanied by lower fitness of resistant populations under insecticide-free conditions compared to susceptible populations^1^,^2^,^3^,^4^,^5^,^6^,^7^,^8^,^9^,^10^,^11^. Moreover, both the magnitude of resistance and the frequency of resistant individuals often decline rapidly in the absence of selection pressure^12^,^13^,^14^,^15^, indicating that the maintenance of resistance incurs direct performance costs, most likely through higher metabolic rate and re-allocation of resources from somatic growth and reproduction to sustain elevated resistance mechanisms^4^,^16^,^17^.

Environmental conditions including the nutritional quality of available food impact physiological body condition which in turn affects performance^18^,^19^,^20^. The quality of the available diet may furthermore interact with heritable life-history strategies^21^ and genetic adaptations to environmental hazards^22^. For example, diets that support good body condition typically also support higher immune response and survival upon pathogen exposure^18^,^23^, and animals in good body condition would similarly be expected to better resist and detoxify secondary metabolites and insecticides. Although it is well known that nutrient availability may play an important role in the ability of insects to handle and detoxify xenobiotics^24^,^25^,^26^,^27^, the interactive effects of nutritional condition and insecticide resistance on life-history outcomes have, to our knowledge, not been investigated.

The German cockroach (*Blattella germanica* L.) is an obligate pest in the anthropogenic environment and populations have been targeted with a multitude of control measures, most prominently including various insecticides. In response, German cockroach populations have evolved an array of physiological and behavioral mechanisms of insecticide resistance^28^,^29^,^30^,^31^,^32^,^33^, and often associated life-history costs have been found^1^,^34^,^35^,^36^,^37^. We used recently collected German cockroaches with moderately high resistance to indoxacarb to investigate interactive effects between nutritional condition and resistance to insecticide on life-history characteristics. Indoxacarb is a pro-insecticide which functions as a sodium channel blocker upon metabolic transformation into toxicologically active metabolites^38^. Resistance to indoxacarb has been associated with higher production of esterase enzymes that presumably break down the active components^39^,^40^,^41^,^42^, and sodium channel mutations that reduce target sensitivity to indoxacarb metabolites^39^,^43^,^44^. Life-history costs of evolving indoxacarb resistance have been reported only in moths^6^,^45^ and a homopteran^15^.

We compared performance parameters among three German cockroach populations with different levels of indoxacarb resistance raised under rich and poor nutritional conditions. We also investigated survival of moderately resistant cockroaches exposed to indoxacarb after completing development on either diet. We hypothesized that the cost of adaptation to the insecticide would be more evident under poor nutritional conditions, and that insecticide resistance and poor nutrition would interactively elevate fitness costs. We furthermore hypothesized that nutritional condition alone would significantly affect the susceptibility of cockroaches to the insecticide.

## Methods

### Populations

Insecticide-resistant and -susceptible strains of German cockroaches originated from two field-collected populations. The insecticide susceptible strain (Orlando Normal; ON) was collected more than 60 years ago in Orlando, Florida and has been maintained as a standard, unselected laboratory population with no known resistance to any insecticides^46^,^47^. Insecticide-resistant cockroaches (PR-712) were collected in Carolina, Puerto Rico in August 2012, at a location where multiple insecticides failed to control the population^47^. Upon collection, this population (*n* ≈ 300 individuals) was allowed to breed over six months to a population size of more than 3000 individuals and then separated equally into two laboratory-maintained populations: one that was selected with indoxacarb over 2.5 years, and one that was maintained unselected with no further exposure to insecticide. We selected for indoxacarb resistance by provisioning Advion gel bait (Syngenta, Greensboro, NC, USA, containing 0.6% indoxacarb) over three consecutive days every two months. This regimen selected cockroaches approximately every generation. Rearing and maintenance of all three populations were in all other aspects similar to conditions described for population cultures in Jensen *et al*.^48^, including rich nutritional conditions. All three populations were maintained at population sizes of 1500–4000 individuals. Density was regulated by removing and replacing one of three corrugated cardboard rolls, harbouring up to one third of the population, at three-month intervals if populations were above 3000 individuals. After 2.5 years of recurrent artificial selection, the three populations showed highly distinct levels of resistance to indoxacarb matching their adaptive backgrounds^47^. All phases of the experiment were performed at 25 ± 2 °C and a 12:12 h L:D regime.

### Diets

We produced nutritionally rich and poor diets with either high or low nutrient concentration based on ground and sieved rat chow (Purina 5001 Rodent Diet; PMI Nutrition International, St. Louis, MO, USA), which is a complete diet of high nutritional quality for growth in German cockroach nymphs^49^. The specific macronutrient composition of Purina rat chow is given in Shik *et al*.^50^. Nutrient-rich diet consisted of rat chow in 4% agar, and nutrient-poor diet consisted of rat chow diluted 1:1 by mass with crystalline α-cellulose (Sigma C-6429, Sigma-Aldrich, St. Louis, MO, USA), also in 4% agar. The rat chow was ground and sieved through a 0.71 mm mesh. Cellulose served as a bulking agent and has very limited nutritional value^51^. Both diets were mixed in distilled water at 50 °C after boiling the water with the agar, and freeze-dried. Diets were provided in biscuit-like cubes of up to 1 cm^3^ and resupplied as needed to ensure constant availability. Diluting with cellulose causes higher consumption as nymphs attempt to compensate for the low nutrient content^51^,^52^, but this nutritional compensation is low when the level of dilution is high. Since larger amounts of nutrient-poor than nutrient-rich diet were consumed, the nutrient-poor diet was resupplied more frequently and in larger cubes than the nutrient-rich diet.

### Effects of population and diet on life-history parameters

Nymphs from each of the three populations were allocated to transparent glass jars (10 cm diameter × 10 cm height) in groups of 10 nymphs per jar within 24 h of hatching (*n* = 30 jars per population and diet). The jars contained a piece of egg carton for harborage and were covered with paper towel squares held in place with rubber bands. The inner walls of the jars were lined with a thin layer of petroleum jelly and mineral oil mixture to prevent climbing. Nymphs had free access to water provided in cotton-plugged glass tubes (9 mm diameter × 75 mm length) that were continuously replaced. Newly eclosed adults were recorded and collected within 24 h of emergence, and individual pronotum width was measured with a calibrated eyepiece micrometer in a dissecting microscope.

### Effect of diet on susceptibility to indoxacarb

Upon eclosion to the adult stage, males from the unselected PR-712 population were maintained individually in Petri dishes (100 mm diameter × 16 mm height) with a water tube and *ad libitum* access to the same diet as during development. At eight days post-emergence, the males were briefly anesthetized with carbon dioxide, and 50 μg of indoxacarb in 0.5 μL of acetone was applied topically on the ventral side of the thorax. Individuals were checked daily for survival and recorded as dead (or dying) when unable to stand on their legs. Maximum survival was set at 28 days, which substantially exceeded the period of indoxacarb lethality. We chose to test the effect of dietary condition on indoxacarb susceptibility in males only from the unselected PR-712 population because we expected this population to best represent the original field population, and because very different doses would be required to assay multiple populations and both sexes due to different resistance levels^47^.

### Statistical analysis

Proportional hazard tests (JMP 7.0, SAS Institute Inc., Cary, NC, USA) were used to analyze overall effects of population, diet, and sex on numbers surviving to the adult stage per jar, development time, adult pronotum width, and survival upon topical indoxacarb application in males from the unselected PR-712 population. Multiple comparisons across populations, diets, and sexes were conducted using Kruskal-Wallis tests at a significance level of α = 0.05 (SPSS 22.0, IBM Institute Inc., Chicago, IL, USA). Proportional hazard and Kruskal-Wallis tests were chosen for all analyses because data were generally not normally distributed (Shapiro-Wilk test, *p* < 0.05), and non-parametric analysis was therefore required.

## Results

### Survival to the adult stage

**Figure 1:**
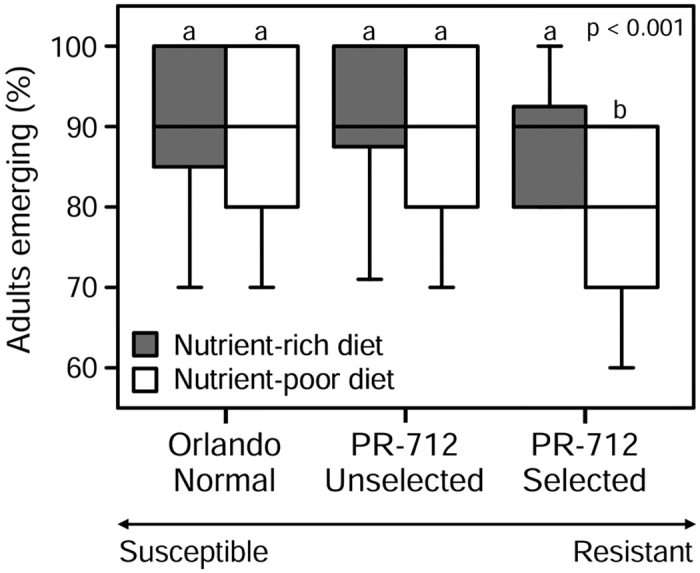
Percentage of individuals surviving development to the adult stage per jar for each cockroach population and dietary condition. Nymphs were set up in groups of 10 per jar within 24 h upon hatching. 30 jars were set up for each population and diet. Boxes show median ± 25^th^ percentiles and 95% confidence intervals. The overall *p*-value is from a Kruskal-Wallis test. Different letters indicate significant differences among populations and treatments at a significance level of α = 0.05 (Kruskal-Wallis post-hoc test).

We found significant effects of both population and diet on survival to the adult stage (Proportional hazard; population: *χ*^2^_2,180_ = 9.7136, *p* = 0.0078; diet: *χ*^2^_1,180_ = 7.8177, *p* = 0.0052), but the interaction effect was not statistically significant (*χ*^2^_2,180_ = 3.8165, *p* = 0.1483). Whereas the majority of nymphs survived to the adult stage across all populations and diets, survival was lower in the indoxacarb-selected PR-712 population than in the two unselected populations, and significantly lower when developing on nutrient-poor diet (Fig. 1).

### Development time

**Figure 2:**
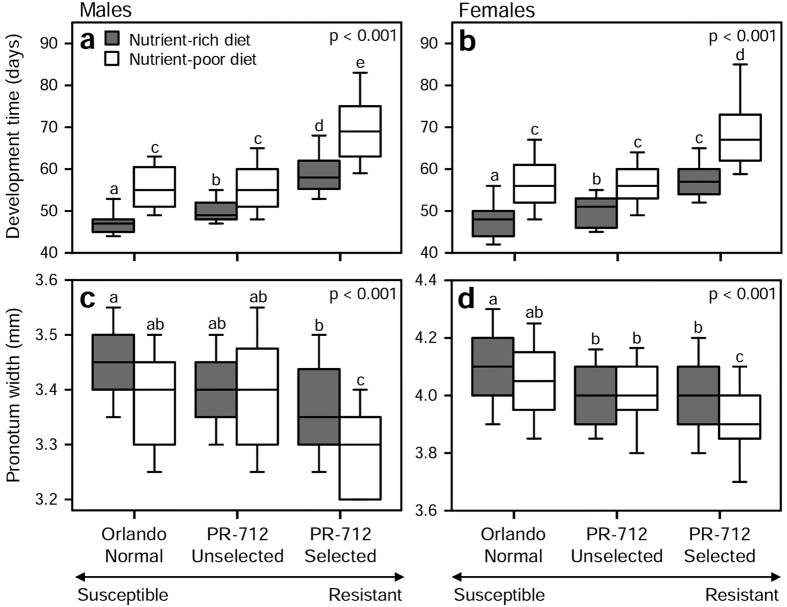
Development time (**a**,**b**) and adult pronotum width (**c**,**d**) in males and females surviving to the adult stage. Nymphs were set up in groups of 10 per jar within 24 h of hatching. Boxes show median ± 25^th^ percentiles and 95% confidence intervals. The overall *p*-values are from Kruskal-Wallis tests. Different letters indicate significant differences among populations and treatments at a significance level of α = 0.05 (Kruskal-Wallis post-hoc tests).

Both population and diet significantly influenced development time to the adult stage; development was prolonged in populations with higher indoxacarb resistance, and poor nutrition prolonged development time within each population (Table 1, Fig. 2a,b). In addition, we found a significant interaction between population and diet on development time (Table 1), indicating that development was further prolonged by poor nutrition in the indoxacarb-selected population and that rich nutrition had a larger positive effect on development rate in the fully susceptible population compared to the moderately resistant population (Fig. 2a,b). We also found a significant interaction between population and sex on development time (Table 1), which might reflect longer median development of males than females in the indoxacarb-selected population, whereas females had longer median development in the other populations (Fig. 2a,b).

### Adult body size

The body size of emerging adults differed significantly depending on both population and diet; pronotum widths were smaller in populations with higher indoxacarb resistance, and poor nutrition generally negatively affected pronotum width within populations (Table 1, Fig. 2c,d). As for development time, we found a significant interaction between population and diet on adult body size (Table 1), reflecting that body size was significantly affected by nutritional condition in the indoxacarb-selected population but not in the other two populations (Fig. 2c,d). We also found a significant interaction between population and sex on body size (Table 1), which might reflect overall larger median size differences between populations in females than in males (Fig. 2c,d).

### Dietary effect on susceptibility to indoxacarb

**Figure 3:**
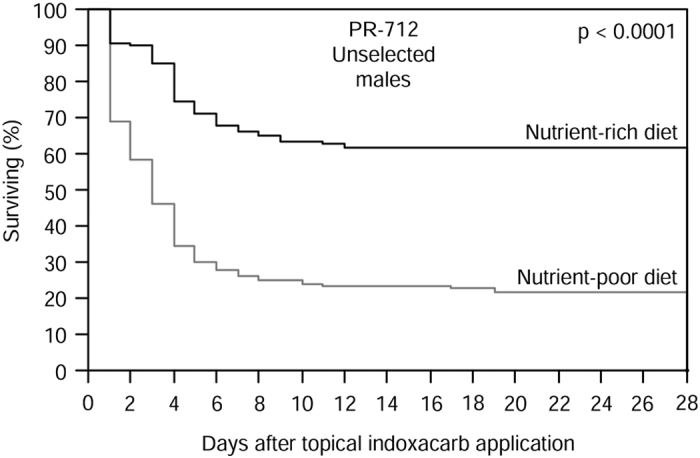
Survivorship curves for PR-712 males from the unselected population after topical application of 50 μg indoxacarb per individual. Newly hatched nymphs were reared to adults in groups of 10 on nutritionally rich or poor diet, after which males were maintained individually in 10 cm Petri dishes with a cotton-plugged water tube and *ad libitum* access to their respective diet. Indoxacarb was applied on day 8 after adult emergence, and survival was recorded daily over 28 days. The *p*-value is from a proportional hazard test.

Susceptibility of unselected PR-712 males to indoxacarb was significantly higher under poor than under rich nutritional condition (Proportional hazard; *χ*^2^_1,256_ = 26.54, *p* < 0.0001; Fig. 3). The majority of males (62%) given nutrient-rich diet survived the indoxacarb treatment, whereas only 22% of the males given nutrient-poor diet survived the insecticide (Fig. 3).

## Discussion

Insecticides impose strong selection pressure on populations to evolve resistance mechanisms to circumvent the hazard, and modify life-history traits as a trade-off to alleviate the costs of resistance^1^,^2^,^6^,^8^,^9^,^10^,^11^. However, the life-history responses of insects to the potentially complex interactions of insecticide resistance with environmental factors such as dietary conditions have been little investigated. We found interactive, reinforcing costs of evolving indoxacarb resistance and undergoing development under poor nutritional condition on individual life-history traits–time to reach the adult stage and adult body size–in German cockroach populations (Table 1, Fig. 2). We also found a significant influence of nutritional condition on survival after direct exposure to the insecticide (Fig. 3).

Survival to first reproduction is arguably the most important determinant of individual fitness. Therefore, absolute survival to the adult stage is not expected to trade-off dramatically with other life-history parameters, while development time and final body size may vary. Our finding that survival to the adult stage was significantly lower in the highly resistant population raised on poor diet indicates that the evolution of high resistance to indoxacarb is associated with severe costs, and that some individuals are not able to compensate for these costs, especially under poor nutritional conditions (Fig. 1). Significantly longer development times in the highly resistant population under both rich and poor nutritional conditions further indicate high costs associated with evolving resistance to indoxacarb (Table 1, Fig. 2a,b), and the very long development time and smaller body size in this population under poor nutritional condition shows that these nymphs in particular struggled to gain enough resources for development and growth (Table 1, Fig. 2). Nevertheless, it is important to note that high insecticide resistance may impose costs on some life-history parameters and not on others. For example, selection with insecticides reduced fecundity and reproductive rate in some bed bug (*Cimex lectularius* L.) populations, but it did not diminish longevity and generation time was shorter in selected populations^10^.

Although highly significant, insecticide resistance and diet had smaller effects on adult body size than on development time (Table 1, Fig. 2), such that adult size was prioritized at the cost of prolonged development. This prioritization was previously shown in *B. germanica*^53^ and more extensively in the moth *Manduca sexta* L.^54^,^55^, indicating that the fitness costs associated with small adult size are higher than costs associated with longer development. In female insects, fecundity typically increases with body size^56^. However, body size usually has little effect on mating success in females, whereas smaller males are at a disadvantage in sexual selection due to lower performance under male-male competition and female choice^57^,^58^. Maturing at a smaller adult size therefore means fewer chances to sire offspring in males while mainly the number of offspring produced per breeding cycle is affected in females. Larger size-dependent fitness consequences in males than in females thus likely explain the interactions between population and sex on development time and body size in our experiment (Table 1).

Diet quality significantly affected the susceptibility of male German cockroaches to indoxacarb (Fig. 3). Various toxicokinetic processes could be affected by diet, including cuticle permeability and metabolic degradation of the insecticide. The availability and quality of nutritional resources likely impact cuticle constituents and the up-regulation of detoxifying enzymes, which are energetically costly to produce and require substantial investment in amino acids and other nutritional components such as sulfur and iron for synthesis^9^,^17^. Higher metabolic rate^4^,^16^ and expenditure of amino acids and micronutrients to sustain a high production of detoxification enzymes likely explain the higher dependence on rich nutritional conditions by the highly resistant cockroach population in our experiment, as resources are allocated to resistance mechanisms instead of growth. If metabolic resistance genes to indoxacarb are identified, it would be interesting to knock down their expression, e.g. by RNA interference, and measure the effect on life-history parameters under variable conditions.

In the moth *Plutella xylostella* L., indoxacarb resistance was found to have arisen twice due to target-site mutations by independent point mutations (F^4i15^Y and V^4i18^I) in genes coding for amino acids within a specific section of the sodium channel^43^. This structure was found to be highly conserved across insects including *B. germanica*^43^, and these same mutations were found to also reduce the potency of indoxacarb when introduced in the sodium channels of this species for functional analysis^44^. It is therefore possible that target-site resistance to indoxacarb in German cockroaches could evolve due to point mutations in the same set of genes. In this context, it would be interesting to determine whether target-site mutations that confer high resistance to insecticides reduce the metabolic investment in detoxification enzymes, which might uncouple the dependence on nutrition to maintain resistance.

In the absence of insecticide, optimal growth can be quickly regained due to natural selection for lower resistance that alleviates the resistance costs^12^,^13^,^15^, and this likely explains the lack of fitness penalty observed after rearing insecticide-resistant German cockroach populations in the laboratory with no insecticide pressure^59^. It is possible that after 2.5 years of laboratory rearing with no insecticide pressure and *ad libitum* feeding conditions, cockroaches in our unselected PR-712 population might have evolved higher developmental performance than upon collection. This population might therefore have adapted to life histories more similar to those of the ON population than when the culture was established, although individuals were still highly resistant when reared under nutritionally rich conditions (Fig. 3).

In the field, nutritional conditions are expected to be suboptimal compared to laboratory conditions^60^, especially if efforts are made to deplete the availability of potential food sources. Paradoxically, laboratory studies examining the effects of insecticides are typically performed under rich nutritional conditions. Although replication with multiple independent populations held at large population sizes will be needed to generalize our conclusions, the present study underscores the substantial influence of nutritional condition on the costs of insecticide resistance to fitness parameters in the absence of insecticide. Our study also showed a large effect of nutritional condition on survival upon direct insecticide exposure. These results emphasize that a key component of insecticide deployment strategies especially when applying toxic bait–minimizing access to alternative food sources–would be particularly effective in controlling populations that are resistant to insecticide.

## Additional Information

**How to cite this article**: Jensen, K. *et al*. Insecticide resistance and nutrition interactively shape life-history parameters in German cockroaches. *Sci. Rep.* **6**, 28731; doi: 10.1038/srep28731 (2016).

## Figures and Tables

**Table 1:** Proportional hazard tests on the effects of population (Orlando Normal, PR-712 unselected, or PR-712 indoxacarb-selected), diet (rich or poor), sex (male or female), and their interactions on development time to the adult stage and on adult body size (pronotum width).

Dependent variable	Factor	*df*	*χ*^2^	*p*
Development time	Population	2	598.90	<0.0001
	Diet	1	455.61	<0.0001
	Sex	1	3.55	0.0596
	Population × diet	2	18.69	<0.0001
	Population × sex	2	11.44	0.0033
	Diet × sex	1	1.19	0.2759
	Population × diet × sex	2	2.34	0.3100
Body size	Population	2	147.61	<0.0001
	Diet	1	37.35	<0.0001
	Sex	1	1993.64	0.0000
	Population × diet	2	28.38	<0.0001
	Population × sex	2	9.04	0.0109
	Diet × sex	1	2.17	0.1405
	Population × diet × sex	2	0.66	0.7172
